# A Monitoring Method Based on FBG for Concrete Corrosion Cracking

**DOI:** 10.3390/s16071093

**Published:** 2016-07-14

**Authors:** Jianghong Mao, Fangyuan Xu, Qian Gao, Shenglin Liu, Weiliang Jin, Yidong Xu

**Affiliations:** 1 Ningbo Institute of Technology, Zhejiang University, Ningbo 315100, China; jhmao@nit.zju.edu.cn (J.M.); sillyliar@sina.com (Q.G.); rui565282919@163.com (S.L.); jinwl@zju.edu.cn (W.J.); xyd@nit.zju.edu.cn (Y.X.); 2 College of Civil Engineering, Chongqing Jiaotong University, Chongqing 400074, China

**Keywords:** Fiber Bragg Grating, reinforced concrete, structural health monitoring, reinforcement corrosion, corrosion cracking

## Abstract

Corrosion cracking of reinforced concrete caused by chloride salt is one of the main determinants of structure durability. Monitoring the entire process of concrete corrosion cracking is critical for assessing the remaining life of the structure and determining if maintenance is needed. Fiber Bragg Grating (FBG) sensing technology is extensively developed in photoelectric monitoring technology and has been used on many projects. FBG can detect the quasi-distribution of strain and temperature under corrosive environments, and thus it is suitable for monitoring reinforced concrete cracking. According to the mechanical principle that corrosion expansion is responsible for the reinforced concrete cracking, a package design of reinforced concrete cracking sensors based on FBG was proposed and investigated in this study. The corresponding relationship between the grating wavelength and strain was calibrated by an equal strength beam test. The effectiveness of the proposed method was verified by an electrically accelerated corrosion experiment. The fiber grating sensing technology was able to track the corrosion expansion and corrosion cracking in real time and provided data to inform decision-making for the maintenance and management of the engineering structure.

## 1. Introduction

One of the main causes of structure durability failure is reinforcement corrosion. The maintenance of corrosive concrete structures costs an estimated $100 billion annually around the world [1]. The occurrence and propagation of reinforcement corrosion deteriorate the usability and safety of reinforced concrete structures. For example, if a bridge was used in a chloride environment for a long period of time, the volume expansion caused by the corrosion of reinforcement could lead to concrete cover cracks that expose the reinforcement to the corrosive environment. Therefore, monitoring reinforcement corrosion is important for guaranteeing the safety of reinforced concrete structures.

At present, commercial sensors based on electrochemical principles, such as the anode ladder sensor [2] and electrode array sensor [3], can also be used to detect reinforcement rusting. In practice, resistance strain gauge is vulnerable to high humidity while the vibrating wires strain gauge is not small enough to embed it into concrete cover. For these reasons, the above sensors cannot discern the extent of the reinforcement corrosion and its influence on the concrete. Optical fiber sensing technology has many advantages, including strong implantation, anti-electromagnetic interference, and good durability. Optical fiber sensing is widely used for monitoring reinforcement corrosion by detecting chloride concentrations [4], pH values [5], and light intensity attenuation [6]. Abu [7] developed an optical fiber corrosion sensor that detects corrosion based on the Bragg reflected wavelength spectra; via an etched-cladding fiber Bragg grating, it can detect the production of corrosion waste. Zheng [8], using FBG sensors packaged with fiber-reinforced plastics (FRP) to wrap around the steel bar, performed accelerating corrosion tests in lab experiments to show that the corrosion sensor was feasible for monitoring the early corrosion of rebar in concrete.

Although these sensors can detect information about the corrosive environment, the detection or monitoring of concrete strain caused by reinforcement corrosion has not been achieved. The Brillouin Optical Time Domain Analysis (BOTDA) [9,10] is a new structural health monitoring technology and can perform multi-dimensional spatial synchronous monitoring in a complex environment. In recent years, BOTDA has also been applied to the monitoring of reinforced concrete durability. The researchers designed the distributed optical fiber sensor monitoring system for the entire process of reinforced concrete corrosion [11] and established the corresponding relationship between the optical fiber strain and the concrete expansion and presence of cracks. However, BOTDA demodulation is costly, limiting its wide use as a durability monitoring sensor.

Compared to BOTDA, Fiber Bragg Grating (FBG) sensing technology has the advantages of high precision and low cost. Therefore, it can be used as an effective method for the monitoring of reinforced concrete corrosion cracking. Lee [12] and Lo [13] proposed a detection method to judge reinforcement rusting; the criterion was collected from a bare FBG sensor pasted on the surface of the reinforcement. They also embedded bare FBG into concrete to determine the corrosion cracking moment and crack location [11]; however, the results were only qualitative. In order to obtain quantitative data, the coefficient between the wavelength and the strain must first be established.

In this paper, packaged concrete corrosion monitoring sensors based on FBG (CCM-FBG) were embedded into concrete. The coefficient between the grating wavelength and strain was calibrated by an equal strength beam test. The effectiveness of the proposed method was verified by an electrically accelerated corrosion experiment. It was found that CCM-FBG was able to track the corrosion expansion and corrosion cracking in real-time and also provided data for decision-making regarding the maintenance and management of the engineering structure.

## 2. Monitoring Principle of Reinforced Concrete Corrosion Cracking Based on FBG

### 2.1. Mechanical Mechanism of Corrosion Expansion and Cracking

Concrete elastically deforms before cracking; therefore, elastic mechanical principles were used to explain the mechanism of corrosion-induced concrete expansion. In this paper, three main assumptions were made:

(1) Elastic deformation: Concrete behaved as an elastic material, such that non-linear deformation was not considered;
(2) Uniform corrosion: Corrosion products uniformly arranged around the steel; therefore, the expansion stress acting on the concrete cover was uniform;
(3) No leakage of corrosion products: Corrosion products did not leak out of the concrete cover; therefore, expansion stress increased with increasing amounts of corrosion products.

**Figure 1:**
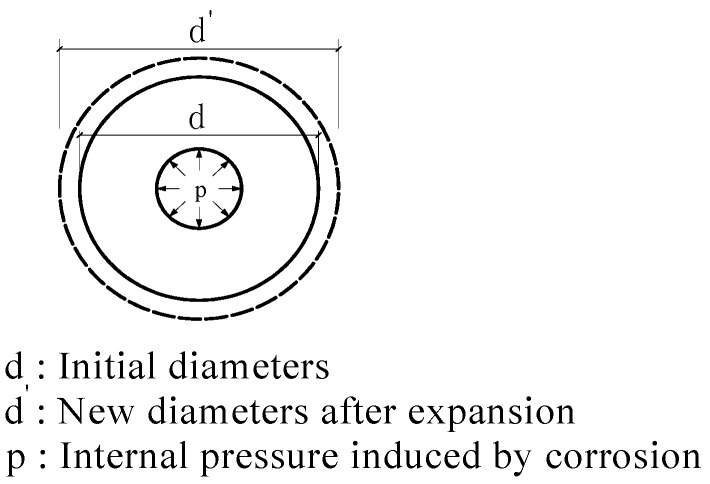
Mechanical model of reinforced concrete corrosion.

The mechanical model of reinforced concrete corrosion is illustrated in Figure 1 [11]:

According to the theory of elasticity, the equation for the uniform internal stress in a circular ring is described in Equations (1)–(3):

(1) $$\sigma =\frac{a^{2}p}{d^{2}-a^{2}}(\frac{d^{2}}{r^{2}}+1)$$

(2) $$\pi d^{\prime }=\pi d(1+\varepsilon )$$

(3) $$\Delta d=d\varepsilon =d\frac{\sigma }{E}=\frac{a^{2}pd}{(d^{2}-a^{2})E}(\frac{d^{2}}{r^{2}}+1)$$

where σ is the hoop stress of concrete, *p* is the internal pressure, *d* is the external diameter of the circular ring, *a* is the internal diameter of the circular ring, *r* is the distance from the analysis point to the centre, ε is the hoop strain at the analysis point; and *E* is the elastic modulus of concrete. From the equations above, it is clear that there is a linear relationship between corrosion-induced expansion (Δ*d*) and concrete strain (ε). Therefore, one can estimate concrete expansion by monitoring hoop strain, which can be recorded by CCM-FBG.

### 2.2. Principle of FBG Sensing Technology

**Figure 2:**
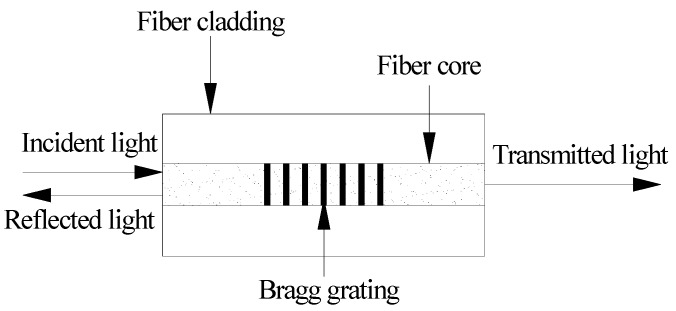
Schematic of FBG sensor technology.

The structure of FBG fiber grating is shown in Figure 2.

FBG is a periodic and permanent modification of the core refractive index value along the optical fiber axis [14]. The FBG sensor operates by measuring the changes in the reflective signal from the grating, and the reflective signal is influenced by the external parameters of the surrounding material. The Bragg wavelength (*λ_B_*) reflected at the sensor is determined by

(4) $$\lambda_{B}=2n_{B}\Lambda$$

where *n_B_* is the reflective index and Λ is the spatial pitch. When projecting a wide spectrum light source onto the optical fibers, the narrow band spectrum, satisfying Equation (4), is reflected back due to the effect of the gratings.

The relationship between the reflective Bragg wavelength shift (Δ*λ_B_*) and changes in strain and temperature in the grating region (*ε_g_*) is expressed as

(5) $$\frac{\Delta \lambda_{B}}{\lambda_{B}}=(1-\rho_{\varepsilon })\Delta \varepsilon_{g}+\left(\alpha +\beta \right)\Delta T$$

where *ρ_ε_* is the effective light pressure coefficient*,* and *α* and *β* represent the thermal expansion and thermo-optic coefficients, respectively. Any changes in external parameters (e.g., temperature, pressure, etc.) alter the grating characteristics and result in a shift of the reflected Bragg wavelength.

### 2.3. Design and Embedding of CCM-FBG

**Figure 3:**
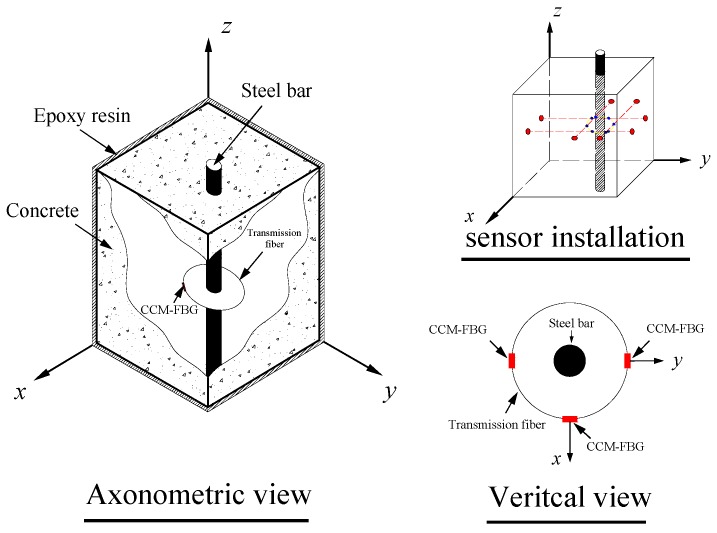
Design and embedding of CCM-FBG.

According to the concrete corrosion cracking mechanism and the FBG sensing principle, a concrete corrosion monitoring sensor, as shown in Figure 3, was designed based on the FBG (CCM-FBG). The FBG sensing units were arranged along the circumference. They were directly connected with the transmission fiber. The FBG sensing units and transmission fiber were fixed on a platform composed of ropes. The ropes were passed through holes in the concrete formwork and then pulled taut to make the platform.

## 3. Packaging and Calibration of CCM-FBG

### 3.1. Packaging Principle of CCM-FBG

It is difficult to guarantee that the bare FBG sensor remains intact and functional after embedding into the concrete. Alternatively, the coefficient could not be established unless the packaging was finished. Thus, a specific packaging process and calibrating test were performed. When embedding CCM-FBG into the concrete, the size of the sensor was a serious consideration. The packaged CCM-FBG needs to meet the following requirements:

(1) Suitable size: In order to avoid stress concentration around the CCM-FBG, the size should be minimized.
(2) Sufficient stiffness: In order to ensure high rate of successful embedding, the CCM-FBG should be sufficiently stiff to resist the impact force during concrete casting.
(3) Temperature compensation: Because the wavelength variation of FBG is also dependent on temperature, the coefficient between the wavelength and the temperature should be determined.

### 3.2. Packaging Process of CCM-FBG

**Figure 4:**
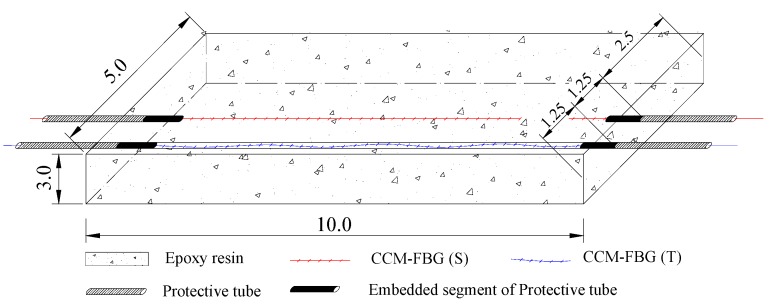
Schematic of the structure of CCM-FBG.

Based on the above principles, the size of the CCM-FBG designed as 10 mm × 5 mm × 3 mm (length × width × thickness). The structure and packaging process of the CCM-FBG are shown in Figure 4. The Bragg grating of the fiber was set at a certain length of the casing and encapsulated in epoxy resin in order to prevent breaks in the bare fiber grating at the free end. Fiber gratings for strain and temperature embedded in the sensor were directly cast into the epoxy resin. The strain fiber grating, marked CCM-FBG (S), was in contact with only a tiny amount of epoxy resin so that the sensor accurately reflected the structural strain. The temperature fiber grating, marked CCM-FBG (T), was kept in a relaxed state in a thin tube so that the sensor was free from structural strain.

### 3.3. Calibration of CCM-FBG

**Figure 5:**
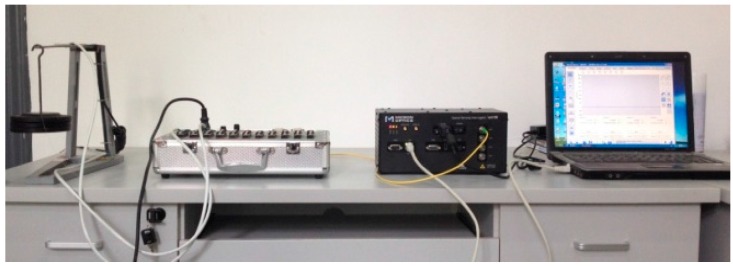
Layout of the calibration test.

An equal strength beam test was performed in order to determine the coefficient between the wavelength and strain. The CCM-FBG and the traditional resistance strain gauges were bonded to the surface of the equal strength beam at the same time. Loads were added at the end of the equal strength beam, 5.0 N each time until 50.0 N was added. CMM-FBG was connected to the SM 130 MOI interrogator, and then, the wavelengths of CCM-FBG were recorded for each load level. As well, the strain of the equal strength beam was recorded by the resistance strain gauges. The layout of the calibration test is shown in Figure 5. The connection between the sensor and equal strength beam is achieved by a thin glue adhesive which can resist high tension force but low shear force. After testing, the sensor can be removed by knocking its lateral side lightly.

#### 3.3.1. Wavelength/Strain Coefficient

**Figure 6:**
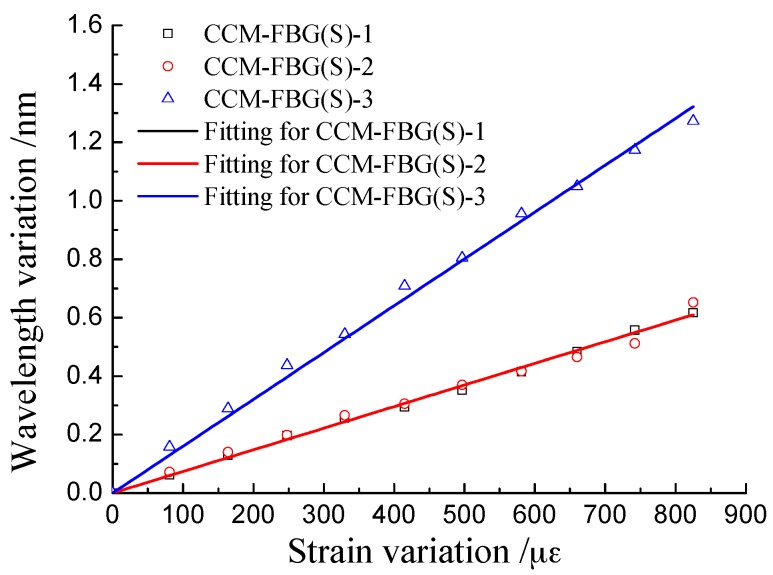
Calibration results of strain for CCM-FBG.

The calibration test results are shown in Figure 6.

As shown in Figure 6, there was a linear relationship between the wavelength variation of CCM-FBG and strain variation. The fittings of the data are shown in Table 1.

The correlation coefficients between the wavelength and strain were close to 1, indicating that the strain of structure can be calculated with high precision from the data of CCM-FBG. The wavelength/strain coefficient of CCM-FBG (S)-1 was twice that of CCM-FBG (S)-2 and CCM-FBG (S)-3. The main reason was that the mixture proportion of resin and curing agent influenced the elastic modulus of the hardened epoxy resin. However, it is difficult to precisely control the mixture proportion.

#### 3.3.2. Wavelength/Temperature Coefficient

**Figure 7:**
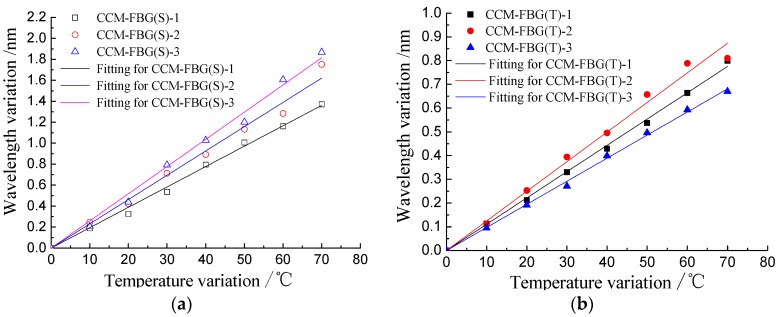
Calibration results of temperature for CCM-FBG: (**a**) CCM-FBG (S); (**b**) CCM-FBG (T).

The wavelength of FBG is also affected by temperature, and thus temperature compensation should be considered for long-term corrosion monitoring. In this paper, the calibration test of wavelength/temperature coefficient of CCM-FBG was conducted. The CCM-FBG was immersed in 80 °C water, and then the temperature was gradually decreased to 10 °C. During this time, the wavelength of CCM-FBG (S) and CCM-FBG (T) was recorded for every 10 °C reduction. These results are shown in Figure 7.

There was a linear relationship between the wavelength variation of CCM-FBG and temperature variation. The data were fitted with a linear regression model, as shown in Table 2.

The correlation coefficients between the wavelength and temperature were close to 1, indicating that temperature compensation could be easily applied. According to the calibration test, the strain caused by the concrete corrosion cracking can be calculated by the following formula:

(6) $$\varepsilon =\frac{\Delta \lambda_{\varepsilon }-\Delta \lambda_{T}\alpha_{\varepsilon }/\alpha_{T}}{\beta_{\varepsilon }}$$

where ε is the strain of CCM-FBG; *Δλ_ε_* is the wavelength variation of CCM-FBG (S); *Δλ_T_* is the wavelength variation of CCM-FBG (T); *α_ε_* is the wavelength/temperature coefficient of CCM-FBG (S); *α_T_* is the wavelength/temperature coefficient of CCM-FBG (T); and *β_ε_* is the wavelength/strain coefficient of CCM-FBG (S).

## 4. Application of CCM-FBG to Concrete Corrosion Monitoring

### 4.1. Experiment Design

Because concrete corrosion in a natural environment occurs over a long period of time, accelerated corrosion achieved with the use of electro chemistry is generally induced for concrete durability research. The evolution process of reinforcement concrete corrosion can still be observed in accelerated corrosion and thus the method was adopted in this study. A steel bar was connected to the positive electrode, and the stainless steel net was connected to the negative electrode of a DC power supply. The specimen was immersed in a 5% NaCl solution. The direct current density was set to 100 A/cm^2^ to ensure slow corrosion so that abundant data was generated for analysis.

The size of the concrete specimen was 150 mm × 150 mm × 150 mm. The concrete strength grade was C30, and the ratio of water: cement: sand: stone was 1:0.52:1.94:3.19. The concrete cover was 30 mm [15] for the steel bar with a diameter of 18 mm. The top and bottom surfaces of the concrete specimen were coated with epoxy resin. The purpose of the epoxy resin coating was to prevent permeation of chloride ions into the interface between the steel bars and concrete; otherwise, the steel bar around the interface would corrode firstly.

**Figure 8:**
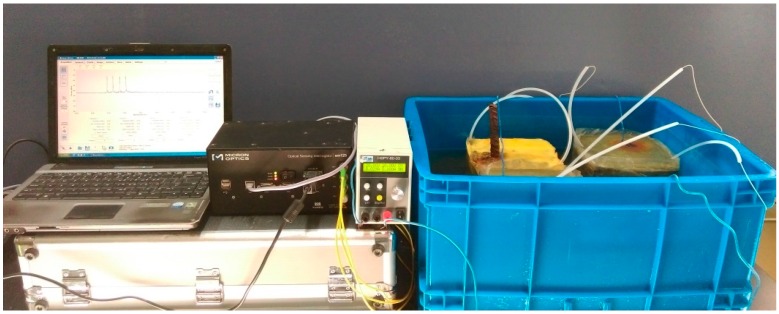
Layout of the accelerated corrosion experiment.

The concrete corrosion cracking due to reinforcement rusting normally occurs at the bottom surface or the lateral side of the RC beam, but the specific location of the cracking is random. Therefore, three FBG sensors were applied to consider the randomness. Three CCM-FBG units were distributed along the reinforcement, and the diameter of the ring was 40 mm. All sensors were connected to the SM 130 MOI interrogator and the data was collected in real-time. The layout of the experiment is shown in Figure 8. The three FBG sensors were independent in this paper, connected to different channels of the interrogator.

The experiment lasted for two months, and the specimen had not been moved during this whole experimental period. In order to observe the surface of the concrete specimen, the NaCl solution was extracted with a small pump regularly every day. With accelerating corrosion, the rust overflowed into the solution along the crack and made the solution turbid and difficult to pump. With the purpose of reducing any disturbance to the corrosive specimen, corrosion products were not cleaned until the end of the experiment.

### 4.2. Monitoring Results of the Accelerated Corrosion Experiment

**Figure 9:**
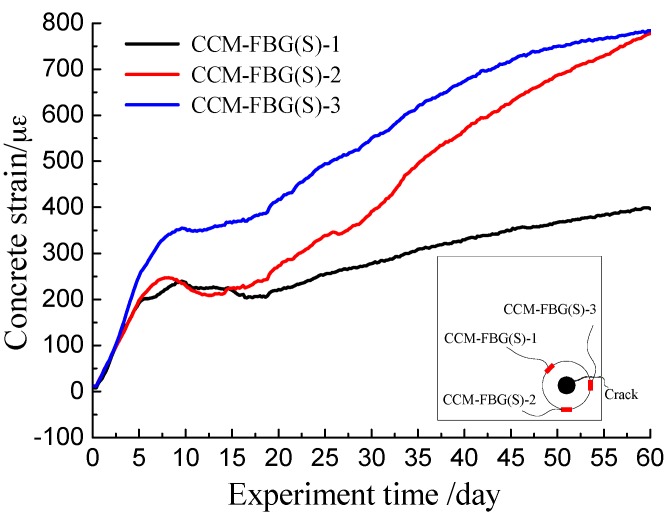
Concrete strain over the course of the accelerated corrosion experiment.

Concrete strain was calculated using Equation (6). The concrete strains recorded by CCM-FBG (S)-1, CCM-FBG (S)-2, and CCM-FBG (S)-3 during the entire experiment are shown in Figure 9. Once the concrete corrosion cracking occurs, the corrosion product outflows from the crack and the rust will turn up at concrete surface. By observing the surface of the beam, it was found that the crack appeared around CCM-FBG (S)-3. The cracks inside concrete were detected by splitting the specimen. After splitting, it was found that the rust was located between CCM-FBG (S)-1 and CCM-FBG (S)-3 (but close to CCM-FBG (S)-3).

As observed in Figure 9, all three CCM-FBG units were able to track the entire corrosion process. Each displayed the same trend of three stages: the expansion stage, the initial cracking stage, and the crack propagation stage. All three CCM-FBG sensors obtained the three particular stages with somewhat different strain values.

(1) Expansion stage.

**Figure 10:**
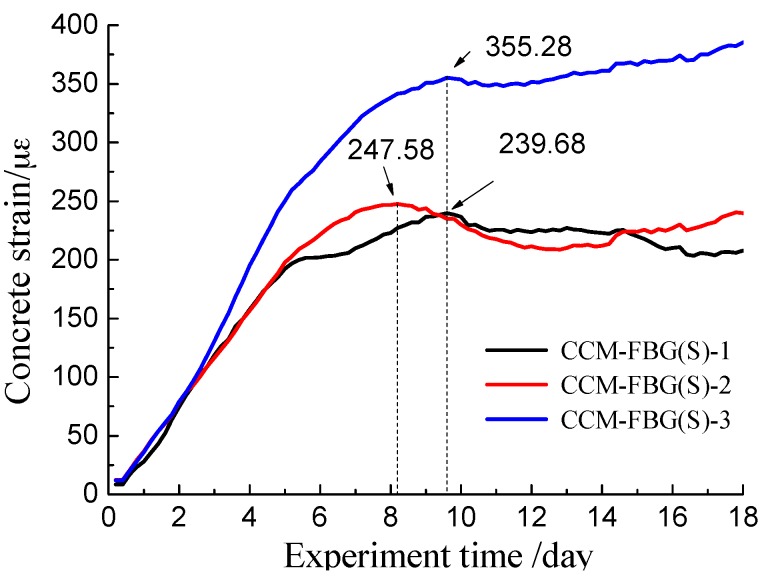
Stage of electrically accelerated corrosion cracking.

This stage lasted about nine days without cracking. The rust kept filling the pore and caused the concrete corrosion expansion force to increase rapidly. The details of strain variations are shown in Figure 10.

During the first three days, the concrete strains obtained by all CCM-FBG (S) were mostly consistent. Then, the increasing concrete strain obtained by CCM-FBG (S)-3 was significantly higher than those recorded by CCM-FBG (S)-1 and CCM-FBG (S)-2. Finally, the maximum strain captured by CCM-FBG (S) occurred at different time. The concrete corrosion crack also appeared near the side of CCM-FBG (S)-3. Alternatively, the corrosion expansion force inside of the concrete was not uniform.

(2) Initial cracking stage.

Once the expansion force of concrete exceeded the tensile strength of concrete, the crack of the concrete appeared at the surface. At the same time, the stress of concrete redistributed and tensile stresses were partially released. As shown in Figure 10, there was a region of decreasing concrete strain. Among them, the concrete strains at CCM-FBG (S)-1 and CCM-FBG (S)-2 significantly decreased, and there was no significant decrease at CCM-FBG (S)-3. The main reason was that there was a crack in the vicinity of CCM-FBG (S)-3. The crack provided a passageway for harmful ions to gather around steel bar, and thus, the relevant high corrosion rate of the reinforcement close to CCM-FBG (S)-3 provided a lasting expansion force.

(3) Crack propagation stage.

Although some corrosion products flowed out during this stage, the filling by the corrosion product still increased the concrete corrosion force; however, the increasing rate was obviously lower than before cracking, as shown in Figure 9. Therefore, the growth rate of the CCM-FBG (S)-1, which was far from the crack, was slower, and the growth rates of CCM-FBG (S)-2 and CCM-FBG (S)-3 were much higher.

## 5. Discussion

(1) The cost of monitoring was low, and the packaging technology was simple.

A kind of concrete corrosion monitoring sensor had been previously developed based on BOTDA [11]. The BOTDA technology was able to obtain the corrosion force and the crack width of concrete. BOTDA could also identify the position of cracking when combined with non-packaged FBG. However, it is difficult for researchers or infrastructure management to use the technology due to its high costs. The sensor developed in this paper is based on FBG. Therefore, this sensor had the advantages of lower cost and easy packaging, both of which can increase application in scientific research and real-projects.

(2) High precision could be beneficial to study and solve scientific problems.

Traditional resistance strain gauges cannot be buried within internal concrete. The BOTDA technology has a relevant lower precision (±20 με); moreover, it is short of high spatial resolution (the minimum spatial resolution is 0.5 m). In this paper, the size of the CCM-FBG was 10 mm× 5 mm × 3 mm, and it could obtain the concrete corrosion force with high precision. The test results show that the concrete corrosion strain behaved heterogeneously in all three stages, and these results asserted the mechanism of the corrosion.

(3) The packaging technology needs to be improved for application in practical engineering.

The simple packaging method was an acceptable approach for short-term research. Once applied to practical engineering, some problems such as service life and material creeping would need to be addressed. Some packaging methods for other sensors based on FBG have already been developed and therefore could be applied to CCM-FBG.

(4) The application of the proposed sensor needs to overcome some challenges.

Although the packaging of the sensor can improve the strength of FBG, the transmission optical fiber cannot be armored and is still brittle; therefore we need to solve the problem of embedding the sensors with low survival. Also, the procedure of embedding the sensor is so complicated that it requires professional staff and a specialized interrogator to obtain data, which is difficult for routing inspection.

## 6. Conclusions and Forecast

Chloride is one of the main causes of failure of reinforcement concrete structures. It is critical to evaluate the remaining service life of structures and make decisions on potential maintenance. In this paper, a CCM-FBG sensor with a size of 10 mm × 5 mm × 3 mm was investigated. The coefficients of wavelength/strain and wavelength/temperature were calibrated. The validity of the CCM-FBG was proved by conducting a two-month accelerated corrosion test. The CCM-FBG accurately reflected the expansion strain, and the real-time monitoring data can be beneficial for determining the mechanism of the corrosion. The technology could be used in practical engineering after further design and improved encapsulation. The sensors provided an effective, long-term, and stable detection and monitoring method of a reinforced concrete structure in a chloride environment. The collected data can inform decision-making for the maintenance and management of engineering structures.

## Figures and Tables

**Table 1:** Simple linear regression model of the wavelength/strain coefficient.

Senor	Wavelength/Strain Coefficient	Correlation Coefficient
CCM-FBG (S)-1	0.0016	0.998
CCM-FBG (S)-2	0.0007	0.996
CCM-FBG (S)-3	0.0007	0.999

**Table 2:** Simple linear regression model of the wavelength/temperature coefficient.

Sensor	Wavelength/Temperature Coefficient	Correlation Coefficient
CCM-FBG (S)-1	0.01944	0.998
CCM-FBG (S)-2	0.02316	0.995
CCM-FBG (S)-3	0.02593	0.997
CCM-FBG (T)-1	0.01107	0.999
CCM-FBG (T)-2	0.01247	0.996
CCM-FBG (T)-3	0.00972	0.999
